# Evaluating the impact of meloxicam oral suspension administered at parturition on subsequent production, health, and culling in dairy cows: A randomized clinical field trial

**DOI:** 10.1371/journal.pone.0209236

**Published:** 2018-12-12

**Authors:** Daniel A. Shock, David L. Renaud, Steven M. Roche, Robert Poliquin, Roger Thomson, Merle E. Olson

**Affiliations:** 1 Agricultural Communications and Epidemiological Research (ACER) Consulting, Guelph, Ontario, Canada; 2 Department of Population Medicine, University of Guelph, Guelph, Ontario, Canada; 3 Solvet Animal Health, Calgary, Alberta, Canada; University of Illinois, UNITED STATES

## Abstract

Parturition is a painful event experienced by cows at the onset of lactation. This pain could lead to a reduced feed intake, altered metabolic and immunological status, and a host of other diseases that could seriously limit her productive herd lifespan. The objective of the current study was to assess the effect of administration of a single dose of oral meloxicam, a non-steroidal anti-inflammatory drug (NSAID), on the production and health status of cows during their lactation. A total of 2,653 (1,009 meloxicam-treated and 1,644 untreated control) cows were enrolled across 20 herds in the provinces of Ontario and Quebec, Canada. Relative to untreated controls, meloxicam-treated cows produced 0.64 kg/day (SE = 0.29. P = 0.03) more milk over the first 3 test days (90–120 days in lactation), had 0.75 times the odds of subclinical mastitis at first test (SE = 0.08, P = 0.01), and were culled or died at 0.46 times the rate (SE = 0.16, P = 0.03) before 60 days in milk. These results are consistent with previous research and lend support to the hypothesis that parturition is a painful event in cattle. Attempts to ameliorate such pain with analgesics is associated with a variety of positive health and production outcomes.

## Introduction

The modern, high-producing commercial dairy cow faces tremendous physiological pressures associated with parturition and subsequent onset of lactation [1]. Specifically, cows transitioning into lactation experience a multitude of interrelated challenges: a reduction in dry matter intake and associated negative energy balance, calcium homeorhesis, stress, various environmental and management challenges, immunosuppression, and pain associated with lactation and calving. Any factor that hinders the cow’s ability to cope with these challenges is associated with an increased risk of metabolic and infectious disease, reduced reproductive performance, lower productivity, and ultimately, shorter herd longevity [2–6]. As the control of pain and disease are necessary components of good animal welfare [7], it is critical to alleviate the pain and inflammation associated with calving. Doing so could have a positive effect on dry matter intakes [8] and inflammation [9], thereby reducing the risk of transition diseases.

The administration of a non-steroidal anti-inflammatory drug (NSAID) could aid in alleviating the negative sequelae occurring because of parturition. Meloxicam is an NSAID that has high oral bioavailability, and acts by inhibiting cyclooxygenase, the enzyme responsible for converting arachidonic acid to prostaglandin [10]. The reduction in prostaglandin, specifically prostaglandin F_2α_, as a result of meloxicam administration at calving could minimize pain and control systemic inflammation [11,12].

Previous research evaluating the administration of NSAIDs orally at parturition has found that treated cows had an increased milk production over their entire lactation [13,14]. However, these trials used tablets that required manipulation prior to administration and thus, a more convenient dosage form may improve practicality for dairy producers. Meloxicam oral suspension (MOS, Alberta Veterinary Laboratories Ltd., Alberta, Canada) is a commercially available suspension that has shown efficacy in treating musculoskeletal pain in cattle [15] and preventing pain and inflammation following band castration [9]. Thus, MOS could represent an alternative to the administration of NSAID tablets at parturition.

The objective of this clinical trial was to evaluate the use of meloxicam administered orally at parturition on production, health, and culling parameters during lactation. We hypothesized that the use of MOS at calving would lead to a reduced level of culling and a higher level of milk production over the entire lactation due to reduced pain and systemic inflammation surrounding parturition.

## Materials and methods

### Ethics statement

The present field-based study was conducted in compliance with the research guidelines set forth by the Canadian Council on Animal Care after appropriate Animal Use Protocol review by the Institutional Animal Care and Use Committee (Alberta Agriculture, Airdrie Alberta, Canada).

### Dairy herds and animals

A convenience sample of dairy herds from the provinces of Ontario (5 herds) and Quebec (15 herds), Canada were recruited for participation in this field-base trial between the months of November 2016 and September 2017. Herds were selected based on: use of a monthly milk recording service (CanWest Dairy Herd Improvement in Ontario and Valacta in Quebec), the predominant milking breed being Holstein, and willingness to adhere to study protocols. The *a priori* goal was to attain 2000 cows (1000 animals per treatment group); however, no formal sample size calculation was made to establish this objective.

### Treatment protocol and data recording

All participating herd owners/managers and their employees were provided with instructions by the study staff outlining the study protocol. Immediately following calving, animals were randomly assigned to receive an appropriate dose of MOS (MOS-group) or remain as untreated controls (UC-group). Randomization was based on farm-level visual cow identification numbers: cows with even numbers received MOS and cows with odd numbers remained as untreated controls. Prior to administration of MOS, study participants were instructed to estimate the weight of the animal and administer MOS (15 mg meloxicam/ml of solution) at an oral dose of 1 mg meloxicam/kg body weight (10 ml MOS/150 kg body weight). As such, study personnel were not blinded to treatment assignment.

Following treatments, participating farm personnel were instructed to record treatment assignment and all pertinent health and disease events (including death and culling) for all cows that had calved after study commencement. Recorded events of interest were dystocia, milk fever, metritis, ketosis, displaced abomasum, mastitis, lameness, culling, and death, with the definitions based on previous research [16]. Dystocia was defined as any calving requiring human intervention. Milk fever was defined as any cow unable to rise within the first 72 hours following calving. A cow was considered to have metritis following parturition if she had abnormal vaginal discharge with or without a reduction in appetence and/or fever (body temperature > 39.5°C). A cow was considered to have clinical ketosis if she had a reduction in appetite accompanied with an increase in ketone bodies in milk, urine, or blood (as measured on the farm). Cows considered to have displaced abomasum had a decrease in appetite accompanied by an audible, high pitched tympanic resonance (ping) from percussion of left or right abdomen between the 9^th^ and 12^th^ ribs. Clinical mastitis was defined as visually abnormal milk (color, reduced viscosity, clots/flakes present) that may or may not be accompanied by signs of localized inflammation (e.g. redness, heat, pain, swelling in the udder) and/or systemic disease (e.g. inappetence, body temperature > 39.5°C). Lameness cases were defined as any cow with a visual limp on inspection. Finally, all cows exiting the herd were required to be recorded, along for their reason for exit (i.e. death, poor production, problem breeder).

### Data collection

Participating herds were enrolled in herd monitoring services (CanWest DHI or Valacta) and submitted a preserved milk sample monthly for the determination of milk constituents (total volume, fat content, protein content, and somatic cell count [SCC]). Production data were then entered into the electronic herd file of participating herds by the monitoring service.

Herd recording data from all cows that calved between the dates of November 2016 and September 2017 were downloaded from the appropriate herd recording software into a comma-separated file (Excel—Microsoft Corporation, Redmond WA) for subsequent joining and analysis. As herds were enrolled on a rolling basis, data was inspected to ensure that all animals included in the analysis corresponded to the date when the farm began participation in the trial. The study period was 1 year in duration, therefore some herds had completed their study participation prior to data download and analysis, whereas other herds were actively enrolling cows. Only the first 3 test days (approximately 40 days between tests) following parturition were included, ensuring the inclusion of a maximal number of animals for analysis.

Variables of interest for analysis included MOS administration recording, date of test day, test day measurements (milk kg, fat and protein %, SCC), lactation number, calving date, dystocia, non-lactating days prior to calving (for mature cows), disease dates, culling/death dates, SCC prior to dry-off of previous lactation (for mature cows).

### Statistical analysis

All datasets were imported as comma-separated files into the STATA 14 statistical software program (StataCorp LP, College Station, TX) for statistical analysis. The cow was considered the unit of analysis for this study.

#### Descriptive statistics

Descriptive statistics were generated for the final dataset. Overall farm demographics were explored and presented. Treatment assignment was assessed comparing animal number (odd/even) versus MOS treatment records. Health, production, and culling data were described. Distributions were visually assessed for all continuous outcomes. For dichotomous variables (e.g. disease and culling), frequency tables were generated. Specific comparisons between MOS and UC cows were made, with univariable statistical comparisons made using Fisher’s exact test, two-sample T-tests, and Kruskall-Wallis one-way analysis of variance where appropriate.

#### Univariable analysis

Statistical comparisons were made between potential predictor variables and outcomes of interest. For this study, outcomes of interest included milk production over the first 3 test days, risk of subclinical mastitis (SCC > 200,000 cells/ml at first test), and risk of culling over the first 60 days in milk. Appropriate univariable regression models were used for each outcome, namely linear (milk) and logistic (risk of subclinical mastitis and culling) models. Variables explored for each model included: treatment group, lactation group (1, 2, and over 3), non-lactating days prior to parturition, year of calving, season of calving, test number (1, 2, 3), dystocia (0 = eutotic calving, 1 = dystotic calving), disease (e.g. ketosis, displaced abomasum), subclinical mastitis (mature cows with SCC > 200,000 cells/ml, first lactation heifers with SCC > 150,000 cells/ml [17]), days in milk at test date, and herd size. Variables that had moderate statistical associations with the outcome of interest (defined at a liberal P-value < 0.2) were included in subsequent initial multivariable models.

#### Multivariable regression analysis

Once preliminary predictors of interest were identified in univariable analysis, appropriate multivariable random-effects regression models were constructed for each outcome of interest.

When assessing test-day milk production, a repeated measures mixed linear regression model was constructed, using random effects to model test number within cow, cow within herd, and herd within province, using a first-order autoregressive correlation structure between time points. When assessing the risk for subclinical mastitis, a random effects logistic regression model was employed, using random effects for cow within herd and herd within province. Due to the relatively rare occurrence of herd exits (culling and death) less than 60 days in milk, a random effects Poisson regression model was constructed, using cow within herd as the random effect. An appropriate offset, corresponding to cow-days contributed was calculated for each individual animal in the study. Briefly, all cows that were culled at less than 60 days in milk contributed only those cow-days in which they were in the herd. This value was generated by calculating the number of days between their calving and herd exit dates. Those cows that did not die and were not culled within 60 days of calving contributed a complete 60 cow-days.

A step-wise backwards elimination process was used for each model, where all those variables identified as potentially associated with the outcome of interest in the initial univariable screening were included in a full multivariable model. Those variables having P-values for partial F-tests or type III tests of fixed effects > 0.05 were eliminated from the model after assessing whether they were part of biologically plausible interaction terms or had a confounding effect on the outcome of interest (e.g. a > 20% change in coefficient values when the term is removed from the model). Continuous variables were assessed for linearity with predicted model outcomes through visually inspecting a LOWESS curve (local weight scatterplot smoothing) for linear relationship, as well as the significance (P < 0.05) of a quadratic term in the model. If a continuous variable did not have a linear relationship with the outcome of interest, it was subsequently categorized based on biologically relevant cut-points, or a quadratic term was retained in the model (if appropriate). Variables retained in the final model were assessed for collinearity through the examination of Pearson or Spearman rank-order correlation coefficients. When high correlation was found between variables (> 0.6), the most biologically appropriate variable was chosen for inclusion in the final model. Standardized residuals were generated and assessed for normality and homoscedasticity for the linear mixed regression model. For the logistic model, fit was assessed by evaluating McKelvey and Zavoina Pseudo-R^2^. Outliers were identified and evaluated using residuals calculated for each model. When an outlier was identified, the animal variables were assessed for biological plausibility. If the animal had biologically implausible variable value (e.g. signifying an entry error), this observation was dropped from the analysis.

## Results

A total of 20 herds were included in the study, comprising 2,653 cows that calved during the study period with at least one test recording (1,664 UC cows and 1,009 MOS cows). Table 1 outlines the general demographics of herds in the study.

**Table 1:** General herd demographics of participating study herds.

Variable	Mean (SD^2^)	Interquartile Range
**Number of Milking Cows**	160 (115)	80–200
**Average Test Day Production**		
**Milk (kg)**	40 (11)	32–47
**Fat (%)**	3.97 (0.77)	3.59–4.60
**Protein (%)**	3.15 (0.36)	2.96–3.38
**SCC**^1^	168 (644)	19–94
**Average Days Dry**	81 (102)	51–66
**Average Days in Milk at First Test**	31 (30)	15–38

^1^SCC = Somatic Cell Count, cells x 1,000 cells/ml

^2^SD = Standard Deviation

When assessing accuracy of MOS administration, there were a total of 36 incorrect treatments (odd-numbered cows that received a dose of MOS) and 347 even numbered animals that did not receive a treatment. These animals were excluded from the analysis, as the study protocol was not adequately followed.

Health-related results and culling/death events are presented in Table 2. Overall, disease recording was quite low and variable between herds. There were 311 total animals culled or dead (~11.7% of all animals calving during the study period), with 121 of these herd exits occurring within the first 60 days in milk. Reasons for exiting the herd are outlined in Table 3 as entered by each producer into their herd management software.

**Table 2:** Disease and death/culling frequency over the study period for participating herds.

Variable	Frequency (# of events)	Percent of Calvings
**Displaced Abomasum**	66	2.5
**Ketosis (clinical)**	95	3.6
**Mastitis**	33	1.25
**Metritis**	10	1.95
**Lameness (clinical)**	33	1.25
**Total Culls and Deaths**	311	11.72
**Culls < 60 days in milk**	121	4.56

**Table 3:** Reasons for a cow exiting the herd over the study period.

Reason	Frequency (# of Events)	Percent of Cows Exiting the Herd
**Died**	13	4.2
**Culled—dairy or beef**	93	29.9
**Low Production**	29	9.3
**Sickness**	2	0.6
**Old Age**	10	3.2
**Injury**	3	1.0
**Mastitis/Udder Problems**	4	1.3
**Lameness**	45	14.5
**Reproduction**	37	11.9
**Unknown**	75	24.1

Associations between MOS treatment and a variety of parameters are outlined in Table 4. Briefly, lactation group differed between the cows enrolled in the MOS and UC groups (P = 0.04); however, the groups were comparable with respect to days dry and season at calving. Both culling and exits < 60 days in milk were lower in MOS versus UC (P = 0.013 and P < 0.0001, respectively). In addition, UC cows had significantly higher SCC at first test relative to MOS cows (683,000 cells/ml versus 356,000 cells/ml, P = 0.001). On univariable assessment, there was also a tendency for test-day milk to be higher in MOS cows relative to UC (42.01 kg vs 41.66 kg, P = 0.2).

**Table 4:** Univariable comparisons of meloxicam-treated and untreated control cows over a variety of pertinent variables.

Variable	UC	MOS	Total	P—Value
**Lactation Group**				0.04
**First**	539	284	823	
**Second**	408	263	671	
**Third and greater**	697	462	1,159	
**Dystocia**	126	74	200	0.75
**Culling**				
**Culls/exits < 60 DIM**	88	33	121	0.013
**Total Exits**	222	89	311	<0.0001
**Displaced Abomasum**	43	23	66	0.59
**Ketosis**	52	43	95	0.14
**Subclinical Mastitis**^1^	683	356	1,039	0.001
**Days Dry—Mean days (IQR**^2^)	83 (51–67)	77 (52–66)	81 (51–66)	0.13
**DIM at first test (IQR)**	32 (15–38)	31 (15–38)	32 (15–38)	0.33
**Linear Score**^3^ **at Dry-Off**	1.58	1.69	1.63	0.40
**Season of Calving**				
**Winter**	390	271	661	0.29
**Spring**	421	237	658	
**Summer**	277	168	445	
**Fall**	556	333	889	

^1^Subclinical mastitis defined as SCC > 200,000 cells/ml (cows) and SCC > 150,000 cells/ml (first lactation heifers)

^2^IQR = Interquartile Range

^3^Linear score = log_2_ (SCC/100) + 3

When assessing the effect of MOS treatment on the first 3 monthly test-day milk production (kg milk/day), after controlling for significant confounders and clustering in time and space, there was a significant increase noted in cows receiving MOS at calving (Table 5). MOS cows produced 0.64 kg per day more milk over the first 3 monthly herd tests than did UC cows (P = 0.03). Lactation group was also significantly associated with milk production, with second and third or greater lactation animals producing 8.66 and 12.83 kg more milk per test than first lactation heifers (P < 0.0001) (Table 5), respectively. Cows experiencing a displaced abomasum produced 7 kg less milk per test than healthy animals (P < 0.0001). Animals with SCC > 200,000 cells/ml on any given test produced significantly less milk than their healthy herdmates as well (Table 5). Cows requiring assistance at parturition also produced 1.7 kg less milk than those cows that calved unassisted (Table 5). Both year of calving and length of the non-lactating period in mature cows also had a significant effect on test-day milk production (Table 5).

**Table 5:** Results of the multivariable repeated-measures mixed linear model assessing the association between MOS treatment and daily milk production (kg) over the first 3 monthly test days.

Variable	Coefficient	SE^1^	P-Value	95% CI^2^
**UC**	Referent			
**MOS**	0.64	0.29	0.03	0.06–1.21
**Lactation Group**				
**1**	Referent			
**2**	8.66	0.52	<0.0001	7.64–9.68
**3**	12.83	0.47	<0.0001	11.91–13.75
**Test Number**				
**1**	Referent			
**2**	0.60	0.30	0.04	-0.02–1.19
**3**	-1.69	0.47	<0.0001	-2.61—(-0.77)
**Displaced Abomasum**	-7.19	0.88	<0.0001	-8.83—(-5.46)
**SCC > 200,000 cells/ml at test date**	-1.59	0.25	<0.0001	-2.09—(-1.09)
**DIM at test**	0.14	0.01	<0.0001	0.12–0.17
**DIM at test (quadratic)**	-0.001	0.00004	<0.0001	-0.0008 –(-0.0006)
**Calving Season**				
**Winter**	Referent			
**Spring**	1.33	0.41	0.001	0.52–2.14
**Summer**	-1.84	0.80	0.02	-3.41-(-0.27)
**Fall**	-1.14	0.84	0.18	-2.79–0.51
**Calving Year**				
**2016**	Referent			
**2017**	-5.27	1.21	<0.0001	-7.65- (-2.89)
**2018**	-5.77	1.65	0.001	-9.02—(-2.59)
**Days Dry**				
**< 60**	Referent			
**60–80**	0.94	0.42	0.02	0.25–1.88
**>80**	0.09	0.45	0.84	-0.80–0.98
**Dystocia**	-1.74	0.55	0.002	-2.81—(-0.65)
**Constant**	34.90	2.01	<0.0001	30.96–38.83

^1^SE = Standard Error

^2^CI = Confidence Interval

Results from the multivariable random-effects logistic regression model are presented in Table 6. Relative to the UC group, MOS cows had 0.75 times lower odds of subclinical mastitis at first test (P = 0.01, Table 6). The McKelvey and Zavoina Pseudo-R^2^ for the model was 0.67. Overall, the predicted probability of subclinical mastitis in MOS cows was approximately 7%-points lower than UC cows (0.33 versus 0.40, respectively). Other variables associated with an increase in odds of subclinical mastitis at first test included lactation group, days in milk at test date, and calving season (Table 6).

**Table 6:** Results of the multivariable mixed logistic regression model assessing the association between MOS treatment and risk for subclinical mastitis (SCC > 200,000 cells/ml) at first test.

Variable	Odds Ratio	SE^1^	P-Value	95% CI^2^
**UC**	Referent			
**MOS**	0.75	0.08	0.01	0.61–0.93
**Lactation Group**				
**1**	Referent			
**2**	0.36	0.06	<0.0001	0.27–0.50
**3**	0.73	0.10	0.03	0.55–0.96
**DIM at first test**				
**< 15 days**	Referent			
**15–30 days**	0.42	0.06	<0.0001	0.31–0.56
**>30 days**	1.23	0.17	0.14	0.93–1.62
**Season**				
**Winter**	Referent			
**Spring**	2.60	0.38	<0.0001	1.95–3.47
**Summer**	4.78	0.73	<0.0001	3.54–6.45
**Fall**	1.40	0.23	0.04	1.02–1.93
**Milk Production**				
**< 40 kg at 1^st^ test**	Referent			
**> 40 kg at 1^st^ test**	2.17	0.11	<0.0001	1.67–2.83
**Constant**	0.28	0.11	0.001	0.12–0.55

^1^SE = Standard Error

^2^CI = Confidence Interval

Results of the multivariable mixed Poisson regression model assessing the association between MOS treatment and risk for culling within the first 60 days following parturition are presented in Table 7. The rate of early exits (<60 days following parturition) in MOS cows was 0.46 times (P = 0.03) that of UC cows, after controlling for confounding (Table 7). Lactation group and milk production at first test were the only other significant covariates included in the model (Table 7).

**Table 7:** Results of the multivariable mixed Poisson regression model assessing the association between MOS treatment and risk for culling or death within the first 60 days in lactation.

Variable	Incidence Rate Ratio	SE^1^	P-Value	95% CI^2^
**UC**	Referent			
**MOS**	0.46	0.16	0.03	0.23–0.92
**Lactation Group**				
**1**	Referent			
**2**	0.21	0.16	0.04	0.05–0.92
**3**	2.25	0.73	0.01	1.19–4.24
**Milk (kg) at first test**	0.90	0.01	<0.0001	0.88–0.93
**Constant**	4.55x10^-27^	2.43x10^-27^	<0.0001	1.60x10^-27^–1.29x10^-26^

^1^SE = Standard Error

^2^CI = Confidence Interval

## Discussion

The results of this study suggest that the administration of meloxicam orally at calving is associated with an increased milk production over the first 3 test days, a decreased odds of subclinical mastitis at first test, and a reduced rate of exits in the first 60 days of lactation. These results are consistent with past work that has assessed the effects that analgesia at calving has on subsequent production and health performance.

### MOS and milk production

Results of the current study are supported by other studies that found a positive milk production response following NSAID administration at calving, though the milk response was not as pronounced as previous studies have noted [9,10,14,15,16]. Conversely, some studies evaluating the effect of analgesia following calving have failed to find an association with increased milk production [17,18]; however, these studies only evaluated daily milk production over the first 2 weeks of lactation; whereas, studies that found associations between meloxicam treatment and daily milk production increases evaluated production over a far longer period of time.

One important difference the current study and any other experimental study evaluating NSAIDS at calving was the broadness in scope. All the studies cited were conducted on one or two dairy farms, with most being university research farms. Conversely, the current study was inclusive of 20 commercial dairy farms across 2 provinces. Such a broad scope increases the external validity of the study; however, based on the extremely low disease recording rates noted in the current study (Table 2), it is possible that any positive effect that meloxicam could have had on milk production was confounded by unmeasured disease occurrences, thus potentially muting the milk production response relative to other research. Inadequate disease recording is a common problem in field-based dairy research. For instance, Koeck et al. [18] found that 40% of all herds enrolled in their study did not keep adequate disease records. When these herds were excluded from the analysis, disease incidences were reported to be 12.6, 3.7, 4.5, 4.6, 10.8, and 9.2% for mastitis, displaced abomasum, ketosis, retained placenta, metritis, and lameness, respectively [18]. Based on these numbers, the only condition that could be considered reliably recorded would be displaced abomasum, with all other levels falling unrealistically lower than that identified in the literature (Table 2).

Previous research has hypothesized that the increase in milk production noted with meloxicam treatment at calving is driven by inhibition of calving-mediated inflammation [19], as inflammatory mediators in mice has been shown to increase apoptosis in milk-producing epithelium [20]. In addition, as meloxicam was found to reduce the incidence of subclinical mastitis at first test, some of this improvement in milk production could potentially be driven by a slight improvement in mastitis cure rates with a concomitant sparing of milk-producing parenchyma from mastitis-associated damage [21]. In addition to anti-inflammatory properties improving milk yield, meloxicam at calving could provide analgesia, subsequently increasing dry matter intakes resulting in milk production improvements. Newby et al. noted that meloxicam-treated cows spend more time-consuming feed (223.4 min/d ± 112.8 SD versus 156.6 min/d ± 73.0 SD, respectively) and had more feedbunk visits (52 ± 56 SD versus 28 ± 13 SD, respectively) relative to placebo-control cows for the first 24 hours following calving [8].

### Meloxicam and subclinical mastitis

NSAIDS are potent anti-inflammatory drugs, capable of muting the pain and inflammation associated with clinical mastitis in dairy cows [22,23]. However, it was surprising to find an impact on the risk of subclinical mastitis and the mechanism for this reduction is unclear. A similar finding was described in a study evaluating treatment with meloxicam in addition to antimicrobial therapy, where the authors noted a reduced SCC of infected cows at subsequent herd tests in those treated with meloxicam and an antimicrobial relative to antimicrobial treatment alone [24]. The current study would support this finding, as cows receiving meloxicam at calving were significantly less likely to have subclinical mastitis at calving, as measured by SCC > 200,000 cells/ml. Recently, ketoprofen was found to have a significant effect on the milk ejection reflex in chronically mastitic cows, with those treated animals experiencing faster milk flows and a reduction in bimodal milk letdowns [25]. Both unit attachment time and bimodal milk letdowns are risk factors for mastitis infections [26]. Parturition causes physiologic and metabolic changes in the body and is associated with a variety of potentially harmful stimuli (e.g. tissue damage, bacterial contamination) that contribute to an increased inflammatory and immune response [27]. Calving stimulates an acute phase response in the cow, as evidenced by an elevation in acute phase proteins (e.g. haptoglobin and serum amyloid A) [27–29]. It is possible that the deleterious effects of unchecked inflammation are attenuated by the selective cox-2 inhibitory activity of meloxicam therapy, thereby facilitating improved immunological responses to infections. This finding requires further research to elucidate the exact mechanism.

### Meloxicam and risk of culling

Treatment with a single oral dose of meloxicam was associated with a significant reduction in culling less than 60 days in milk relative to untreated controls. Previous research, focusing on culling following clinical mastitis episodes, found that the culling risk for meloxicam-treated cows was about half that of control cows [24]. Though the magnitude differs in the current study, the overall trend is similar. There are a variety of factors that could have driven this reduction in culling risk. Cows that produce less milk, have higher SCCs, and are not pregnant are far more likely to be culled from dairy herds relative to high producing, low SCC, and pregnant cows [30]. As MOS was associated with production and SCC, this could be a plausible explanation. An additional explanation of this lowered culling rate could be that MOS cows had significantly greater feed intakes and reduced systemic inflammation, reducing their risk of metabolic and infectious disease. This theory is currently speculative, as it is not possible to assess the relationship between disease incidence and meloxicam treatment due to the highly variable and inadequate levels of disease recording in the herd records. Finally, lactation played a significant role in the rate of culling, with first and third or greater lactation cows exiting the herd at an increased rate relative to second lactation animals. After controlling for the small, though statistically significant, differences in lactation distribution between treatments (e.g. MOS-treated cows had a higher proportion of cows in their 3^rd^ or greater lactation) the relationship between treatment and herd exit remained significantly different. Future studies should focus on the effect that meloxicam has on the subsequent metabolic status and disease incidence of early lactation cows.

### Study limitations

The focus of the current study was to conduct a field trial including as many commercial dairy farms as was practical. In doing so, study personnel likely compromised their ability to monitor disease recording in trial cows. Disease incidence was quite variable between farms and was not reliable to use as outcomes of interest. In addition, it is possible that a significant proportion of variance unaccounted for by the statistical models could be due to lack of attribution (e.g. unrecorded disease events).

Further, this study was not conducted using a placebo-control group. Neither study participants, nor study authors (e.g. statistical analysts), were blinded to experimental treatments. One potential mitigating factor is that milk production and SCC outcomes evaluated were objectively measured by blinded milk recording services. In addition, it seems unlikely that commercial dairy producers would cull cows based on treatment (e.g. these producers were likely not motivated to remove control cows from their herds at a rate that was roughly double that of meloxicam-treated cows).

## Conclusion

A single treatment with oral meloxicam to recently calved cows was associated with an increase in milk production for the first three tests following parturition, a reduction in the odds of subclinical mastitis infections at first test, and a reduction in the risk of leaving the herd through death or culling within the first 60 days following parturition. These results are consistent with previous research and lend credence to the hypothesis that parturition is a painful event in cattle and attempts to ameliorate such pain with analgesics is associated with a variety of positive health and production outcomes.
