# Correction: Evaluating the impact of meloxicam oral suspension administered at parturition on subsequent production, health, and culling in dairy cows: A randomized clinical field trial

**DOI:** 10.1371/journal.pone.0210326

**Published:** 2018-12-31

**Authors:** The PLOS ONE Staff

## Notice of republication

This article was republished on December 21, 2018 to correct an error in the author byline introduced during the typesetting process. The publisher apologizes for the error. Please download this article again to view the correct version. The originally published, uncorrected article and the republished, corrected article are provided here for reference.

## Supporting information

S1 File Originally published, uncorrected article. (PDF)

S2 File Republished, corrected article. (PDF)

## References

[1] Shock DA, Renaud DL, Roche SM, Poliquin R, Thomson R, Olson ME (2018) Evaluating the impact of meloxicam oral suspension administered at parturition on subsequent production, health, and culling in dairy cows: A randomized clinical field trial. PLoS ONE 13(12): e0209236 10.1371/journal.pone.0209236 30540846 PMC6291144

