# Correction: Evaluating the impact of meloxicam oral suspension administered at parturition on subsequent production, health, and culling in dairy cows: A randomized clinical field trial

**DOI:** 10.1371/journal.pone.0229872

**Published:** 2020-02-26

**Authors:** Daniel A. Shock, David L. Renaud, Steven M. Roche, Robert Poliquin, Roger Thomson, Merle E. Olson

Animals that had missing values for somatic cell count (scc) were erroneously coded as “high SCC” and classified as subclinical mastitis cases when, in fact, they should not have been included in the analysis.

In the Abstract, there is an error in the fifth sentence. The correct sentence is: Relative to untreated controls, meloxicam-treated cows produced 0.64 kg/day (SE = 0.29. P = 0.03) more milk over the first 3 test days (90–120 days in lactation), had 0.12 lower log SCC at first test (SE = 0.06, P = 0.05), and were culled or died at 0.46 times the rate (SE = 0.16, P = 0.03) before 60 days in milk.

In the Univariable analysis section of the Statistical analysis subsection of the Materials and Methods, there is an error in the second sentence. The correct sentence is: For this study, outcomes of interest included milk production over the first 3 test days, risk of subclinical mastitis (SCC > 200,000 cells/ml at first test), natural logarithm of SCC at first test, and risk of culling over the first 60 days in milk.

In the Multivariable regression analysis section of the Statistical analysis subsection of the Materials and Methods, there is a missing sentence after the second sentence. The missing sentence is: When assessing the natural logarithm of SCC at first test, a random effects linear regression model was employed, using random effects for herd within province.

In the Results, there are a number of errors in the sixth paragraph. The correct sixth paragraph is: Results from the multivariable random-effects linear regression model evaluating the natural logarithm of SCC at first test are presented in Table 6. Of note, MOS cows roughly 6,000 cells/ml less at first test than UC (Back-transformed SCC of 44,800 cells/ml in MOS versus 50,513 in UC cows, P = 0.05) (Table 6). In addition, UC cows had significantly higher SCC at first test relative to MOS cows (245,000 cells/ml versus 145,000 cells/ml, P = 0.006). Results from the new logistic regression model assessing risk of subclinical mastitis at first test were not significant (Odds Ratio = 0.85, P = 0.27).

There are a number of errors in Table 6 and the caption for Table 6. Please view the correct Table 6 here.

**Table 6:** Results of the multivariable mixed linear regression model assessing the association between MOS treatment and the natural logarithm of somatic cell count at first test.

Variable	Coefficient	SE^1^	P-Value	95% CI^2^
**UC**	Referent			
**MOS**	-0.12	0.06	0.05	-0.24–0.00
**Lactation Group**				
**1**	Referent			
**2**	-0.07	0.09	0.41	-0.25–0.10
**3**	0.37	0.09	<0.0001	0.19–0.54
**Season**				
**Winter**	Referent			
**Spring**	0.10	0.08	0.24	-0.06–0.26
**Summer**	0.14	0.09	0.15	-0.04–0.32
**Fall**	0.15	0.09	0.07	-0.02–0.31
**Days in milk at 1**^**st**^ **test**				
**<15**	Referent			
**15–30**	-0.49	0.08	<0.0001	-0.64 –(-0.33)
**>30**	-0.59	0.08	<0.0001	-0.75 –(-0.42)
**Milk at first test**				
**< 40 kg**	Referent			
**> 40 kg**	-0.51	0.07	<0.0001	-0.66 –(-0.35)
**Constant**	11.24	0.11	<0.0001	11.03–11.47

^1^SE = Standard Error

^2^CI = Confidence Interval

In the Meloxicam and subclinical mastitis subsection of the Discussion, there are a number of errors in the first paragraph. The correct paragraph is: NSAIDS are potent anti-inflammatory drugs, capable of muting the pain and inflammation associated with clinical mastitis in dairy cows [22,23]. Although there was no influence on subclinical mastitis incidence at first test, there was a modest reduction in SCC. A similar finding was described in a study evaluating treatment with meloxicam in addition to antimicrobial therapy, where the authors noted a reduced SCC of infected cows at subsequent herd tests in those treated with meloxicam and an antimicrobial relative to antimicrobial treatment alone [24]. The current study would support this finding, as cows receiving meloxicam at calving had modestly lower SCC at first test. Recently, ketoprofen was found to have a significant effect on the milk ejection reflex in chronically mastitic cows, with those treated animals experiencing faster milk flows and a reduction in bimodal milk letdowns [25]. Both unit attachment time and bimodal milk letdowns are risk factors for mastitis infections [26]. Parturition causes physio- logic and metabolic changes in the body and is associated with a variety of potentially harmful stimuli (e.g. tissue damage, bacterial contamination) that contribute to an increased inflammatory and immune response [27]. Calving stimulates an acute phase response in the cow, as evidenced by an elevation in acute phase proteins (e.g. haptoglobin and serum amyloid A) [27–29]. It is possible that the deleterious effects of unchecked inflammation are attenuated by the selective cox-2 inhibitory activity of meloxicam therapy, thereby facilitating improved immunological responses to infections. This finding requires further research to elucidate the exact mechanism.

In the Conclusion, there is an error in the first sentence. The correct first sentence is: A single treatment with oral meloxicam to recently calved cows was associated with an increase in milk production for the first three tests following parturition, a modest reduction in SCC at first test, and a reduction in the risk of leaving the herd through death or culling within the first 60 days following parturition.

## References

[1] Shock DA, Renaud DL, Roche SM, Poliquin R, Thomson R, Olson ME (2018) Evaluating the impact of meloxicam oral suspension administered at parturition on subsequent production, health, and culling in dairy cows: A randomized clinical field trial. PLoS ONE 13(12): e0209236 10.1371/journal.pone.0209236 30540846 PMC6291144

